# Control of Cytocompatible Metallic and Polymeric Wrinkle Morphologies Using Programming via Printing (PvP)

**DOI:** 10.1021/acsomega.5c10402

**Published:** 2026-01-05

**Authors:** Johnson N. Agyapong, Teng Zhang, James H. Henderson

**Affiliations:** † Bioinspired Syracuse: Institute for Materials and Living Science, 2029 Syracuse University, Syracuse, New York 13244, United States; ‡ Department of Mechanical and Aerospace Engineering, Syracuse University, Syracuse, New York 13244, United States; § Department of Biomedical & Chemical Engineering, Syracuse University, Syracuse, New York 13244, United States

## Abstract

Mechanical instability-driven wrinkling of a thin rigid film on a compliant substrate, such as a shape-memory polymer (SMP), offers spatiotemporal control of surface topography and morphology. Current strategies for creating wrinkles on SMPs often rely on mechanical actuation achieved via costly or complicated uniaxial or multiaxial loading rigs. There is a need for an accessible and easily democratizable strategy that can produce both simple aligned wrinkles and complex two-dimensional (2D) postbuckling patterns. Using a hobbyist three-dimensional (3D) printer, here we employed a recently developed single-step SMP fabrication approach, programming via printing (PvP), to prepare preprogrammed shape-memory substrates capable of uniaxial or biaxial contraction of varying magnitudes, with the strain controlled by the nozzle temperature and number of orthogonal layers within the substrate. Samples without an orthogonal layer (all filaments are parallel) produced a maximum uniaxial contraction along the same axis as the extruded filament (up to 40%) and underwent Poisson expansion along the perpendicular direction (up to 15%). The substrates were coated with either gold or polystyrene, and shape-memory-driven change was triggered to cause mechanical instability-driven wrinkling of the thin film. To characterize the relationship between orthogonal layers and wrinkle morphology, wrinkles were imaged at the microscopic level using atomic force microscopy (AFM). The transition from unidirectional to bidirectional contractile strain generated by the orthogonal layers was observed to induce a transition from simple aligned wrinkles to complex 2D postbuckling patterns. C3H10T1/2 cells showed high viability (>90%) irrespective of wrinkle morphology 24 h post seeding. Collectively, the results demonstrate a convenient fabrication strategy for producing both simple aligned wrinkles and complex 2D postbuckling patterns, including cytocompatible wrinkles suitable for cell studies.

## Graphical abstract

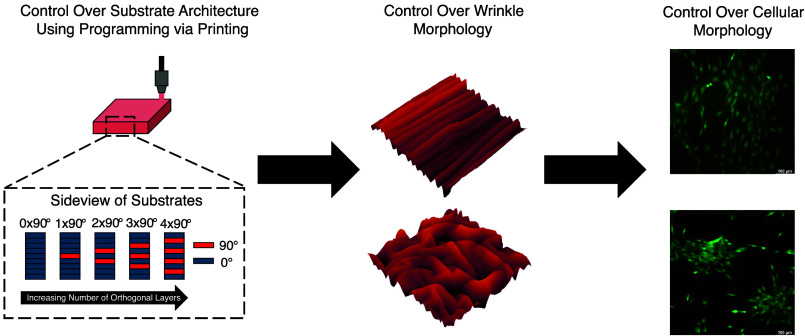

## Introduction

Mechanical instability-driven wrinkling is a surface functionalization strategy that offers spatiotemporal control of surface topography and morphology for applications in metrology, particle sorting, biomimicry, tissue engineering, and cellular mechanobiology.^1–6^ This wrinkling phenomenon occurs in a bilayer system composed of a soft, compliant substrate and a thin, stiff film when the contraction of the compliant substrate exceeds a critical compressive strain, causing buckling of the thin film.^4,7^ The resulting wrinkles can have a simple or complex morphology, depending on the magnitude and direction of the compressive strain imposed on the thin film by the compliant substrate. Here, we use the term “simple” wrinkle morphologies to refer to anisotropic one-dimensional wrinkles, such as uniaxial or striated wrinkles, and “complex” postbuckling patterns or morphologies to refer to two-dimensional surface patterns (examples include radially oriented, ring-shaped, labyrinth-shaped, and crumpled wrinkles) or wrinkles with multiple wavelengths, such as hierarchical wrinkles.^7–11^

Mechanical instability-driven wrinkling has the potential to be a straightforward and cost-effective alternative to other surface functionalization strategies, such as soft lithography.^4,12^ Soft lithography begins with the design of a pattern, followed by the fabrication of a mask, master, and elastomeric stamp.^13^ The design of the pattern and the fabrication of the mask and elastomeric stamp are straightforward processes that can be achieved under ambient laboratory conditions. However, master fabrication is a costly process since the primary techniques involved are e-beam lithography (EBL) and photolithography.^13^ EBL is a slow process that requires expensive equipment and an experienced operator. Photolithography requires a clean room, which is not readily available to most laboratories and institutions. Even when clean rooms are available, extensive training is required to gain access, which is time-consuming. Relative to soft lithography, wrinkling is simple and inexpensive, since it can be achieved under ambient laboratory environments. Furthermore, if combined with functional materials, such as shape-memory polymers (SMPs), wrinkling can be triggered to occur dynamically *in situ*, making it ideal for biomimetic applications and bioinspired designs.^4,10,14–16^

Despite the progress, exciting opportunities to improve mechanical instability-driven wrinkling exist. Most notably, existing approaches for deforming the compliant substrate, whether a passive material or a functional material, such as an SMP, frequently rely on mechanical actuation achieved via costly or complicated uniaxial or multiaxial loading rigs. For example, uniaxial deformation is commonly achieved using a uniaxial loading frame.^4,14,15^ This approach is facilitated by widespread access to uniaxial loading frames but can be used to achieve only simple, aligned wrinkles. In contrast, the formation of complex two-dimensional (2D) postbuckling patterns is a more mechanically involved process that generally requires more complex instrumentation to produce bidirectional or localized strain. Bidirectional strain requires customized clamps but can produce labyrinth-shaped and crumpled wrinkles.^9,12^ Localized strain programming can be achieved through the indentation of a compliant surface using steel balls or a spherical indenter^8^ and can produce radially oriented and ring-shaped wrinkles at the areas of indentation.

To both broaden the potential use cases of mechanical instability-driven wrinkling and facilitate its adoption as a surface functionalization strategy, there is a need for a universally available strategy that can produce both simple aligned wrinkles and complex 2D postbuckling patterns. The goal of the present study was to develop and characterize a wrinkling strategy based not on mechanical actuation by loading rigs but on three-dimensional (3D) printing and programming of SMPs. Using a hobbyist 3D printer, we employed a recently developed single-step SMP fabrication approach, programming via printing (PvP), to prepare preprogrammed shape-memory substrates capable of uniaxial or biaxial contraction of varying magnitudes. To demonstrate use with materially and methodologically different thin films, the substrates were coated with either gold or polystyrene, and shape-memory shape change was triggered to produce simple wrinkles and complex 2D postbuckling patterns. The tunability and robust control over wrinkle morphology were demonstrated by characterizing wrinkles by using optical microscopy and atomic force microscopy (AFM). Lastly, the cytocompatibility of these wrinkles was characterized to assess their potential use in biomedical applications.

## Materials and Methods

### Experimental Overview

To achieve a practical fabrication strategy that can produce both simple wrinkles and complex 2D postbuckling patterns, our strategy was to use programming via printing (PvP) a 4D printing strategy based on melt-extrusion 3D printing of SMPs.^17–19^ In PvP, the process of extruding, drawing, and depositing the SMP filaments is used to introduce tensile strains into each extruded filament, hence programming strains while printing the substrates.^17^ To facilitate systematic modification of surface patterns, substrate architecture and uniaxial or biaxial shape-change behavior was controlled by varying ply orientation (Scheme 1). To demonstrate use with materially and methodologically different thin films, PvP substrates with varying ply orientations were coated with a thin, rigid gold (Au) or polystyrene (PS) film and heated to 70 °C to undergo shape recovery. The shape recovery of the PvP substrate introduced the compressive strain required to buckle the thin film, thereby forming wrinkles. The wrinkles were characterized by AFM to determine the effect of substrate architecture on wrinkle morphology, and the cytocompatibility of the simple wrinkles and complex 2D postbuckling patterns were characterized using a live/dead assay.

**Scheme 1:**
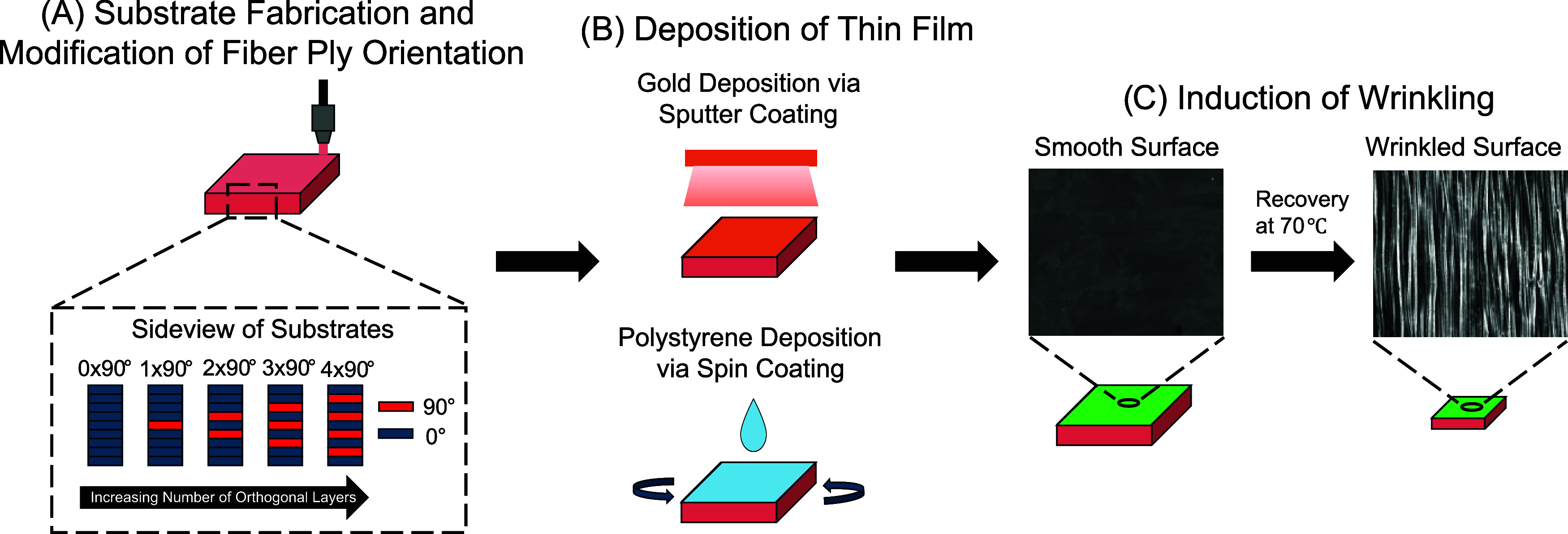
Experimental Overview Detailing the Substrate Fabrication Process^a^

a (A) The process begins with the modification of fiber ply orientation. (B) The printed substrate is then coated with a thin rigid film (Au or PS). (C) The surface is initially smooth and subsequently becomes wrinkled when recovered.

### Substrate Fabrication

Spools of filament were prepared from MM3520 pellets (SMP Technologies Inc., Tokyo, Japan), a commercial thermoplastic polyurethane SMP, as previously described.^18^ Briefly, the extruder used was a 3Devo Composer 350 (3Devo B.V., Utrecht, The Netherlands). An Ender-3 melt-extrusion printer (Creality 3D Technology Co., Ltd., Shenzhen, China) was then used to print 100% infilled square substrates with a length and width of 10 mm. Each substrate was composed of 10 layers, each with a 0.2 mm layer thickness, resulting in a ∼2 mm total substrate height. File preparation for printing was as previously described.^18^ To ensure surface smoothness, the build plate was covered with a strip of Kapton tape, and the first layer was printed very close to the build plate. This eliminated the gaps between the fiber-wielding points, as shown by the smooth surface observed after the introduction of the thin film (Scheme 1). Because the first layer was compressed in this way, it was not considered in the count of ply layers. To control bidirectional strain, we systematically varied filament ply orientation within the remaining 9 layers of the printed substrates (Scheme 1). These layers were modified to have 0, 1, 2, 3, or 4 orthogonal layers. The layers were arranged symmetrically in an alternating pattern starting from the middle layers to minimize bending (Scheme 1). All samples were printed at two nozzle temperatures (200 and 220), to facilitate comparison to previous studies that establish the relationship between the nozzle temperature and strain and elucidate the synergistic effect of the nozzle temperature and orthogonal layers on strain.

### Analysis of Trapped Strain

The modification of the filament ply orientation was expected to affect the magnitude and direction of the trapped strain. The strain (ε) was characterized as the percent change in length upon recovery using eq 1, where *L*_f_ = final length after recovery and *L*_i_ = initial length before recovery.

1 $$\varepsilon \;\left(\%\right)=\frac{L_{i}-L_{f}}{L_{i}}\times 100$$

The strain was characterized for the *x* and *y* directions of each substrate. The *x*-axis represents the 0° orientation, and the *y*-axis represents the 90° orientation.

### Deposition of the Thin Rigid Films

To demonstrate use with two materially and methodologically different thin, rigid films, both were explored in this study, with each illustrating the use of a different deposition strategy. The PS thin film was coated onto the surface of the substrates by using a spin coater (WS-650-23B, Laurell Technologies, Lansdale, PA). To prepare PS for spin coating, PS pellets with a reported molecular weight of 210,000 g mol^–1^ (GPC) (Scientific Polymer Products Inc., Ontario, NY) were dissolved in toluene at 2 or 3 wt % (wt %). The solution was then coated onto the surface at 3000 rpm for 1 min as previously described.^3^ The PS wt % was varied by changing the weight of PS dissolved in toluene to achieve different film thicknesses. The toluene evaporated during spinning, leaving behind the PS film. This allowed the study of the effect of PS thickness on the wrinkle properties. The Au thin film was sputtered onto the substrates using a Vacuum Desk V sputter coater (Denton Vacuum, Moorestown, NJ) at 45 mA for 40 or 100 s. The sputter time was systematically increased to prepare different Au film thicknesses. This allowed the investigation of the effect of the Au film thickness on wrinkle properties.

### Film and Wrinkle Characterization

Film thickness and wrinkle properties were characterized by using atomic force microscopy (AFM, Nano-R2, Pacific Nano Technology, Santa Clara, CA). The AFM was in contact mode with a 5 N m^–1^ spring constant tip. To measure film thickness, Au and PS films were deposited on a glass slide, and film thickness was measured using a scratch assay, as previously described.^4,20^ Using Gwyddion 2.60 (Department of Nanometrology, Czech Metrology Institute, Jihlava, Czechia) to analyze the AFM profiles, the step height from the scratched region to the intact film was measured as the film thickness. Gwyddion was also used to process and analyze the AFM profiles of the wrinkled films. First, the AFM profiles were leveled using the mean plane subtraction tool, and background noise was removed using the remove polynomial background tool. All of the processed images were shifted to a minimum value of zero. Next, line profiles were extracted from the AFM profiles. Using the peak find tool in Gwyddion, wavelength was calculated as the average distance between the two peaks and the amplitude as half of the average height of each peak. Lastly, wrinkle morphology was quantified by calculating wrinkle directionality using the directionality plugin in ImageJ (v. 1.54f., Bethesda, MD, https://imagej.net/ij/). The plugin computed the Fourier transform of the input AFM image to determine the amount or count of structures within a given direction as a histogram, which depicts the preferential orientation of the structures within that image. Each AFM profile presented in this study is a representative image of three technical replicates.

### Live/Dead Cell Assay

Samples for the live/dead assays were cut into 4 mm × 4 mm squares and sterilized for at least 30 min using a biological safety cabinet’s built-in 365 nm UV bulb (Thermo Fisher Scientific, Waltham, MA). Next, samples were equilibrated in Basal Medium Eagle (BME) supplemented with 10% fetal bovine serum, 2 mM l-glutamine, and 1% penicillin-streptomycin for at least 30 min to allow nonspecific protein adsorption. C3H10T1/2 murine fibroblast cells (ATCC, Manassas, VA) were expanded in the BME to passages 5–10. Cells were seeded at 400 cells mm^–2^ for experiments and incubated (37 °C, 5% CO_2_) for 24 h. Finally, a live/dead calcein-acetoxymethyl (Calcein-AM, Invitrogen, Manassas, VA), ethidium homodimer-1 (EthD-1, Invitrogen) viability assay was performed following the manufacturer’s instructions.

### Statistics

One-way analysis of variance (ANOVA) with a Holm–Sidak post hoc test was used to analyze the statistical significance (*p* < 0.05) of the relationship between film thickness and wrinkle dimensions. Welch’s unequal variance *t* test was used to analyze the effect of Au sputter time and PS concentration on film thickness. All statistical analyses were conducted using OriginLab data analysis software (OriginLab v. 9.8.5.212, Northampton, MA).

## Results and Discussion

### Characterization of Trapped Strain

Samples were printed at two different nozzle temperatures to characterize the effect of nozzle temperature on strain which is well studied in previous work.^18,19,21^ To highlight the relationship between strain and nozzle temperature and allow a fair comparison to previous work, the maximum trapped strain of samples with zero orthogonal layers was characterized. As the nozzle temperature increased from 200 °C (Figure 1, left) to 220 °C (Figure 1, right), the maximum trapped strain was observed to decrease from 40.90 ± 0.75% to 18.00 ± 0.27%. These findings match previous studies, which established that, as nozzle temperature increases, trapped strain is expected to decrease.^18,19,21^ The strain decreased as nozzle temperature increased because polymer molecules extruded a higher nozzle temperatures have more time to reorganize into energetically favorable orientations,^22,23^ hence decreasing trapped strain.

**Figure 1:**
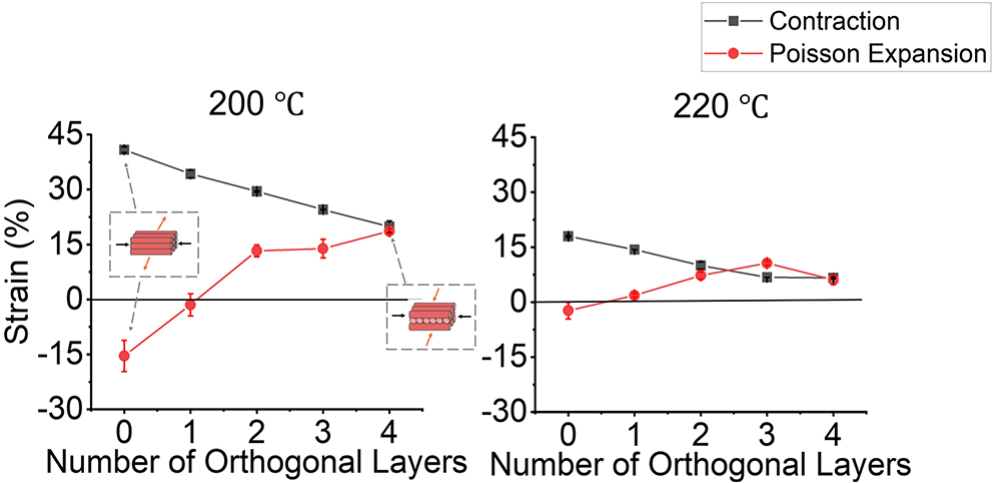
Effect of orthogonal layers on trapped strain at 200 (left) and 220 (right) nozzle temperatures. On these graphs, positive values denote contraction and negative values denote expansion. At both nozzle temperatures, the number of orthogonal layers affects the magnitude and direction of trapped strain. A substrate composed of zero orthogonal layers (all filaments are parallel) produced a maximum contraction along the same axis as the extruded filament and undergoes Poisson expansion along the perpendicular direction. The introduction of orthogonal layers leads to a decrease in both contraction and Poisson expansion, tending toward biaxial strain as the ratio of orthogonal layers approaches unity. The inset demonstrates the Poisson effect at zero orthogonal layers and biaxial contraction at four orthogonal layers. Each point is an average of three technical replicates, and the error bars (which are comparable in height to the data markers for many points) represent the standard deviation.

The samples composed of zero orthogonal layers also served as a benchmark for understanding the effect of orthogonal layers on substrate deformation. In the zero orthogonal layer samples, uniaxial contraction was observed in the fiber direction, and Poisson expansion in the orthogonal direction. As would be expected, when compared to samples containing orthogonal layers, the zero orthogonal layer substrates produced the maximum contractile strain and Poisson’s expansion observed for both nozzle temperatures (Figure 1). Upon the introduction of one orthogonal layer, the contractile strain and Poisson’s expansion in the perpendicular direction decreased regardless of nozzle temperature (Figure 1). The decrease in strain continued as more orthogonal layers were added due to the orthogonal layers introducing a contractile strain perpendicular to the original fiber direction that competes with the contractile strain in that primary direction. As more orthogonal layers are introduced, the secondary contractile strain increases, thus competing with and decreasing both the contraction and Poisson’s expansion observed in the unconstrained zero layer samples. As the ratio of orthogonal layers approaches unity, biaxial contraction is achieved. For this case, the conservation of sample volume during contraction is maintained not by the Poisson expansion in the x or y direction but in the z direction, which, unlike strains in the x or y direction, has no effect on wrinkle formation.

### Effect of Orthogonal Layers on Wrinkle Morphology

The transition from unidirectional to bidirectional contractile strain generated by the orthogonal layers was observed to induce a transition from simple aligned wrinkles to complex 2D postbuckling patterns. To characterize the relationship between orthogonal layers and wrinkle morphology, wrinkles were imaged at the microscopic level using AFM. Afterward, wrinkle morphology was quantified using directionality histograms. As mentioned above, nozzle temperature affects trapped strain, and this effect extends to how orthogonal layers affected the wrinkle morphology of both films. At high trapped strains (samples printed at 200 °C), simple wrinkles were observed on the Au (Figure 2) and PS (Figure 3) surface of substrates composed of zero (Figures 2 and 3, columns 0) and one orthogonal layers (Figures 2 and 3, column 1). In contrast, complex 2D postbuckling patterns were observed on the surfaces of substrates composed of two or more orthogonal layers (Figures 2 and 3, columns 2–4) regardless of film thickness. This behavior was also confirmed by directionality histograms of each orthogonal layer condition (Figures 2C and 3C). For simple wrinkles, a normal distribution centered around 0° was observed (Figures 2C and 3C, columns 0 and 1), while a more even distribution was observed for complex 2D postbuckling patterns (Figures 2C and 3C columns 2–4).

**Figure 2:**
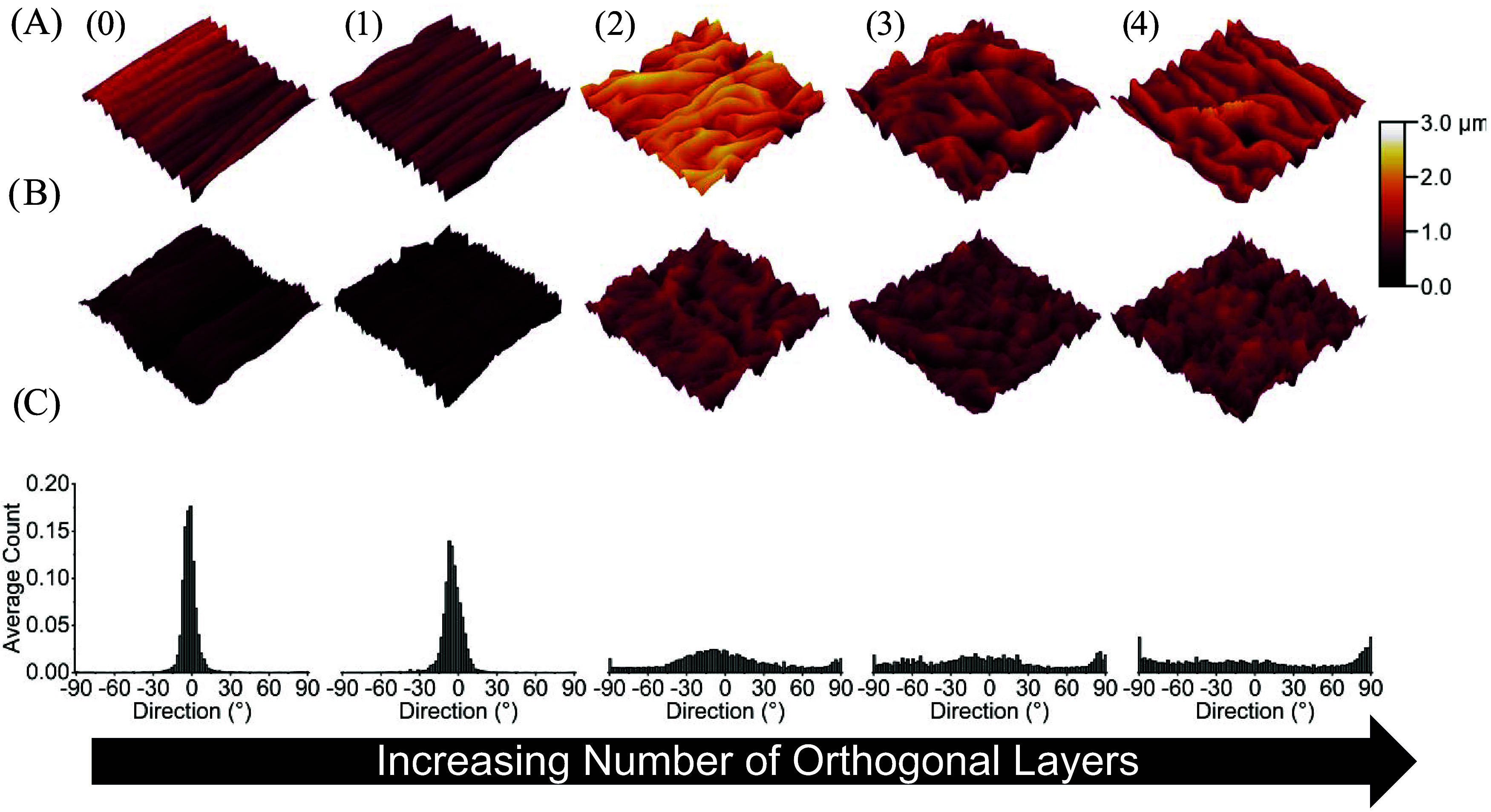
3D AFM profiles and directionality histograms of Au wrinkles on the surfaces of substrates printed at 200 °C. (A) Representative 3D AFM profiles of substrates sputtered for 100 s, which yielded a thickness of 21.63 ± 0.98 nm. (B) Representative 3D AFM profiles of substrates sputtered for 40 s which yielded an Au thickness of 8.95 ± 0.21 nm. (C) Average directionality histograms of wrinkle topographies on the surface of Au coated samples. Samples were composed of zero (0), one (1), two (2), three (3), or four (4) orthogonal layers. Each AFM profile is a representative image of three technical replicates. The dimensions of each AFM profile are 20 μm × 20 μm and the color correspond to the height of surface features. Each histogram is an average of the directionality histograms of three AFM images per orthogonal layer condition.

**Figure 3:**
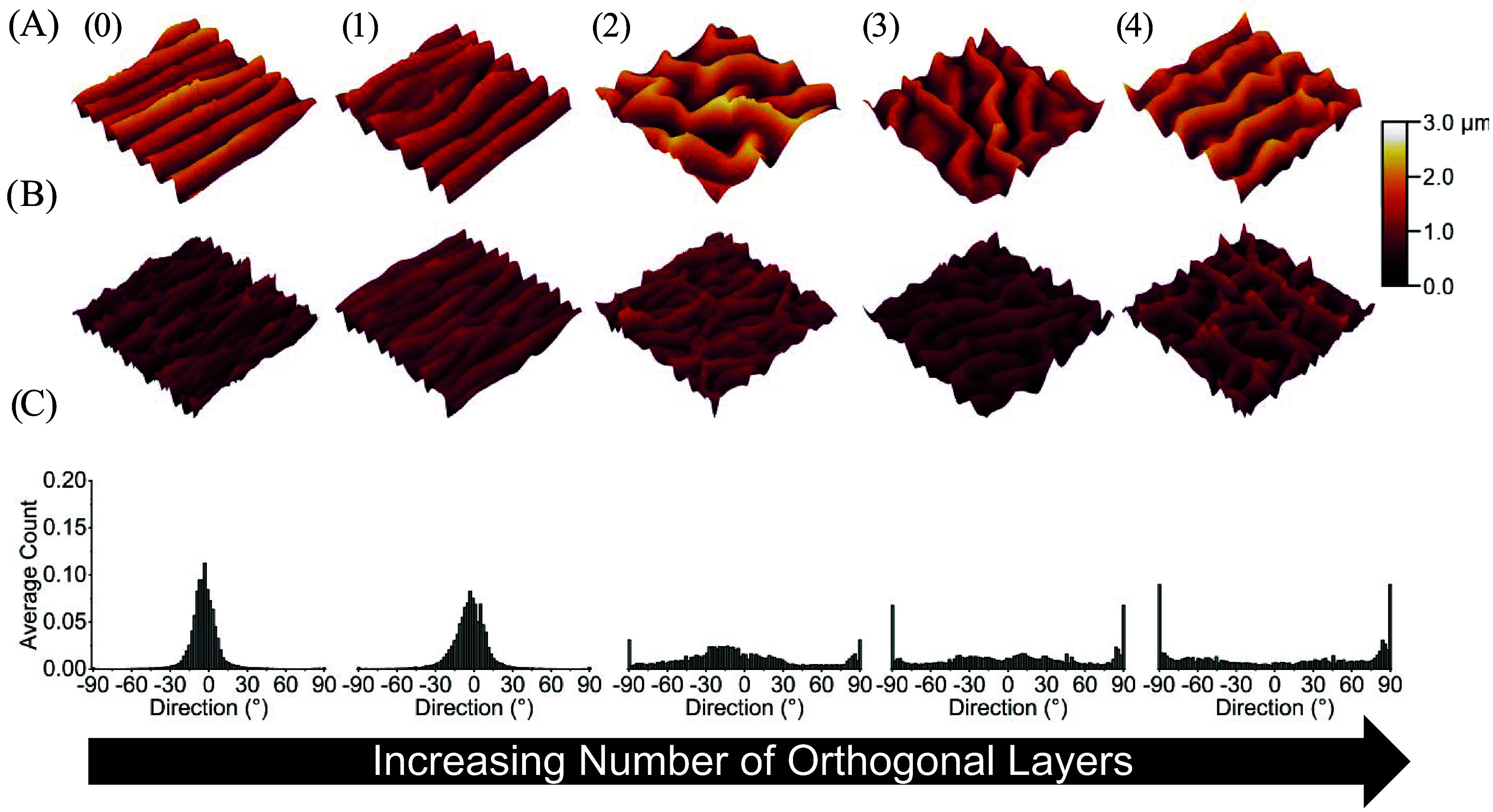
3D AFM profiles and directionality histograms of PS wrinkles on the surfaces of substrates printed at 200 °C. (A) Representative 3D AFM profiles of substrates coated with 3 wt % PS which yielded a PS thickness of 284.92 ± 7.88 nm. (B) Representative 3D AFM profiles of substrates coated with 2 wt % PS which yielded a PS thickness of 90.47 ± 2.95 nm. (C) Average directionality histograms of wrinkle topographies on the surface of PS coated samples. Samples were composed of zero (0), one (1), two (2), three (3), or four (4) orthogonal layers. Each AFM profile is a representative image of three technical replicates. The dimensions of each AFM profile are 20 μm × 20 μm and the color correspond to the height of surface features. Each histogram is an average of the directionality histograms of three AFM images per orthogonal layer condition.

To understand the effect of the orthogonal layer on wrinkle morphology, we reviewed the literature reports that controlled wrinkle morphology via other approaches. Lin and Yang demonstrated experimentally that wrinkle morphology on the surface of PDMS can be controlled by manually releasing unidirectional or bidirectional strains produced by uniaxial or biaxially loaded frames.^24^ In that study, the unidirectional and bidirectional strains produced simple aligned wrinkles and complex 2D postbuckling patterns, respectively. This behavior has since been confirmed to occur on the surfaces of conventionally programmed SMPs as well.^8,9,12,16^ In SMP substrates, the transition from simple aligned wrinkles to complex 2D postbuckling patterns was shown to be dependent on the ratio of strain along the planar axes.^12^ In the present study, the introduction of orthogonal layers tuned the ratio of trapped strain along the orthogonal axes (Figure 1), which in turn controlled the simple wrinkles and complex 2D postbuckling patterns observed (Figures 2–5).

**Figure 4:**
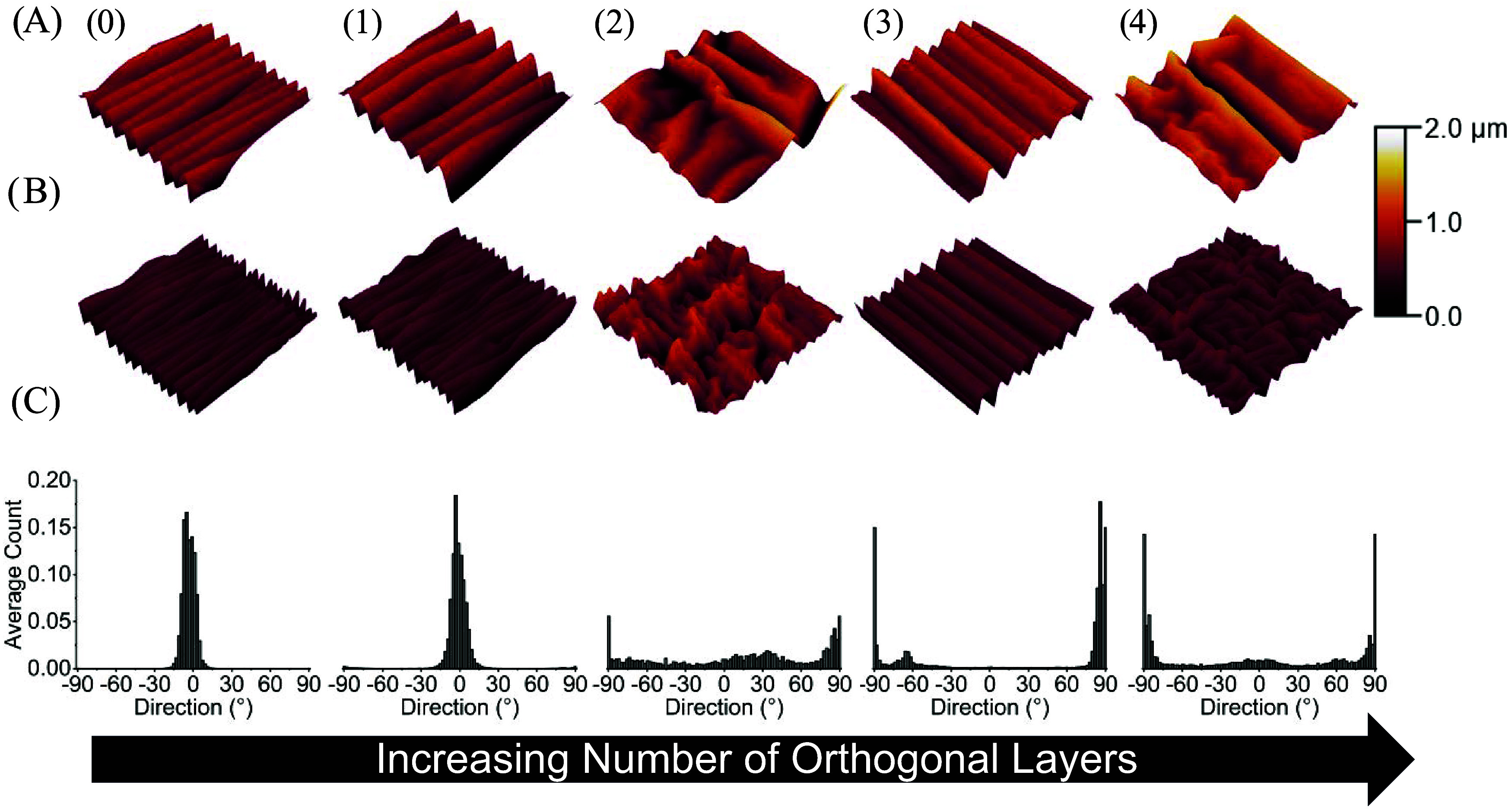
3D AFM profiles and directionality histograms of Au wrinkles on the surfaces of substrates printed at 220 °C. (A) Substrates sputtered for 100 s which yielded a thickness of 21.63 ± 0.98 nm. (B) Substrates sputtered for 40 s which yielded an Au thickness of 8.95 ± 0.21 nm. (C) Average directionality histograms of wrinkle topographies on the surface of Au coated samples. Samples were composed of zero (0), one (1), two (2), three (3), or four (4) orthogonal layers. Each AFM profile is a representative image of three technical replicates. The dimensions of each AFM profile are 20 μm × 20 μm and the color correspond to the height of surface features. Each histogram is an average of the directionality histograms of three AFM images per orthogonal layer condition.

At low trapped strains (substrates printed at 220 °C nozzle temperature), the transition from simple aligned wrinkles to complex 2D postbuckling patterns as a function of orthogonal layers was observed to be unstable for both films (Figures 5 and 6). Here, instability refers to the absence of a clear relationship between the orthogonal layers and wrinkle morphology. For example, the wrinkled Au (Figure 5) and PS (Figure 6) films were observed to have simple wrinkles at zero and one orthogonal layers. At two orthogonal layers, complex Au and PS wrinkles were observed; however, simple Au and PS wrinkles oriented in the 90° direction dominated when three or four orthogonal layers was introduced (Figures 4 and 5, columns 3 and 4). Even though simple wrinkles dominated the three and four orthogonal layer conditions, some samples within these groups produced complex 2D postbuckling patterns (Figure 4A,B column 4 and Figure 5B column 3).

**Figure 5:**
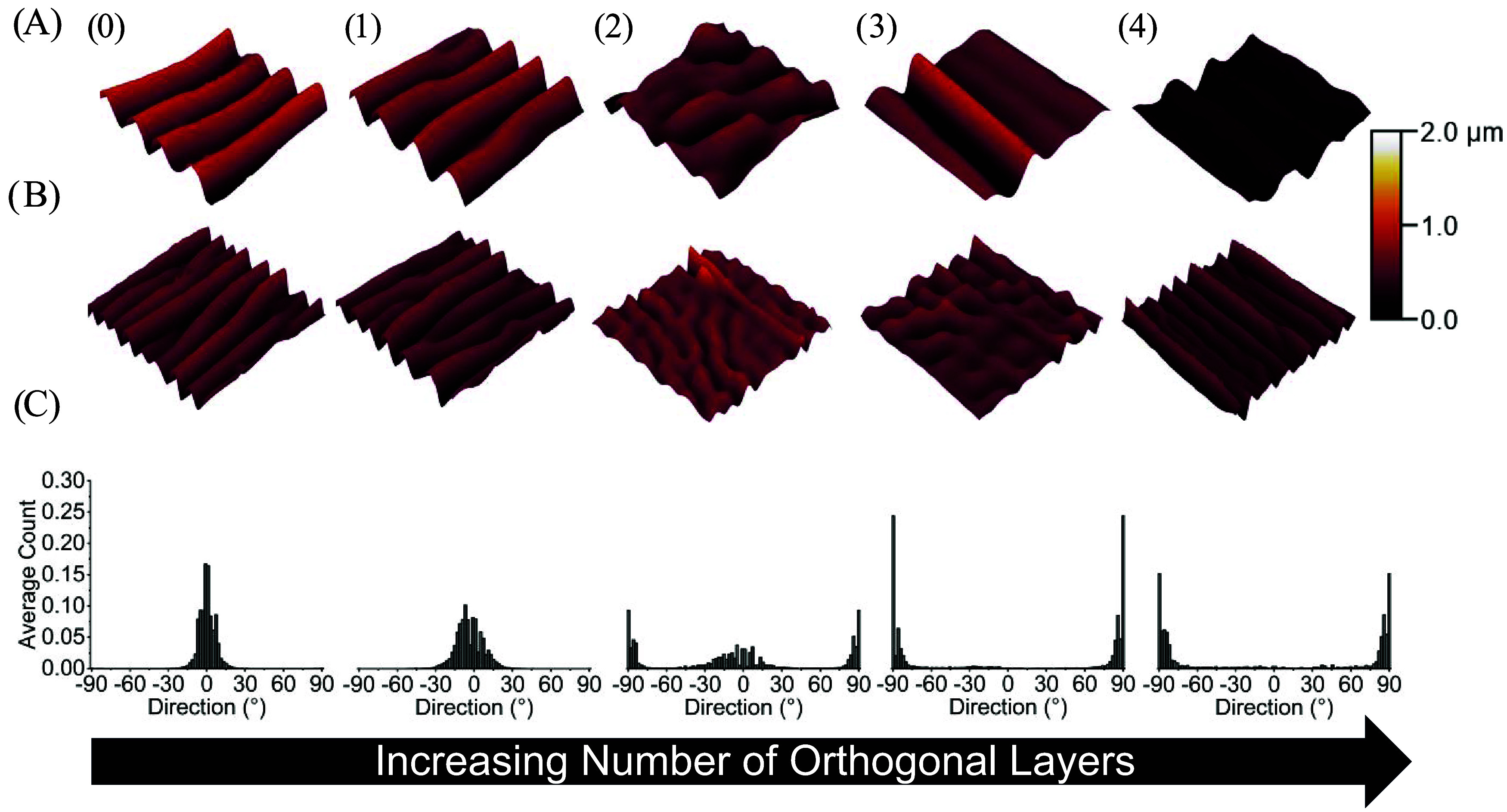
3D AFM profiles and directionality histograms of PS wrinkles on the surfaces of substrates printed at 220 °C. (A) Substrates coated with 3 wt % PS which yielded a PS thickness of 284.92 ± 7.88 nm. (B) Substrates coated with 2 wt % PS which yielded a PS thickness of 90.47 ± 2.95 nm. (C) Average directionality histograms of wrinkle topographies on the surface of PS coated samples. Samples were composed of zero (0), one (1), two (2), three (3), or four (4) orthogonal layers. Each AFM profile is a representative image of three technical replicates. The dimensions of each AFM profile are 20 μm × 20 μm, and the color correspond to the height of surface features. Each histogram is an average of the directionality histograms of three AFM images per orthogonal layer condition.

**Figure 6:**
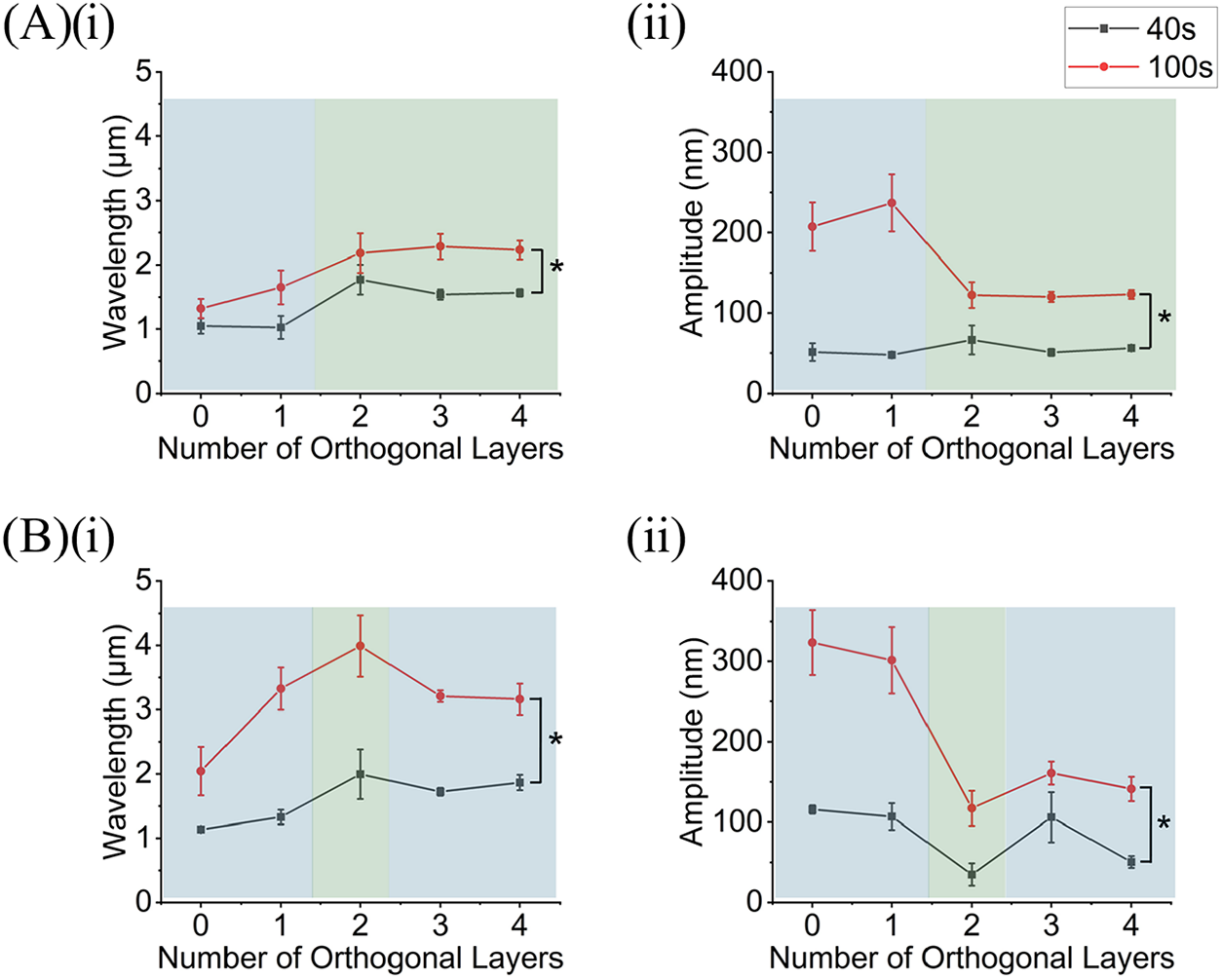
Wavelength and amplitude of Au wrinkles. (A) Wavelength (i) and amplitude (ii) of Au wrinkles on the surface of substrates printed at 200 °C. (B) Wavelength and amplitude of Au wrinkles on the surface of substrates printed at 220 °C. The blue highlight denotes simple wrinkle regime, and the green highlight denotes the complex 2D postbuckling patterns regime. Each point corresponds to an average of three samples, and the error bars represent their standard deviation. All data were compared using one-way ANOVA with Holm–Sidak post hoc test (* *p* < 0.05).

The lack of a clear relationship between the orthogonal layers and wrinkle morphology in the low-strain samples can be explained by the critical strain. The critical strain (ε_c_), according to the linear buckling theory, refers to the threshold of strain above which wrinkle formation can be induced.^4,7,15^

$$\varepsilon_{c}=\frac{1}{4}({\frac{{\mathrm{E̅}}_{s}}{{\mathrm{E̅}}_{f}})}^{2/3}$$

In the ε_c_ equation, *E̅* represents the plane strain modulus, which is related to the Young’s modulus (*E*) and the Poisson’s ratio (*v*) as follows *E̅* = *E*/(1 – *v* ^2^). *E̅*_s_ and *E̅*_f_ represent the substrate and film, respectively. Using the ε_c_ equation, the derived critical strain of PS was 0.438%, and Au was 0.054%. The critical strain of the PS film is an order of magnitude higher since the Young’s modulus of Au (≈78 GPa) is an order of magnitude higher than the Young’s modulus of PS (≈3.5 GPa).

Samples printed at a higher nozzle temperature were characterized as low strain, because their strain is low relative to the critical strain required to induce buckling. In the Au samples with low strains, the unstable transition from simple aligned wrinkles to complex 2D postbuckling patterns (Figures 4 and 5) can be attributed to the contractile strains being close to the critical strain. Specifically, in the biaxial cases the contractile strains produced along one or both axes are likely right around or below the critical threshold, leading to inconsistent morphologies. In this PS condition, this behavior is more apparent. Smooth regions were identified among the PS wrinkles as the number of orthogonal layers increased (Figure S6, see Supporting Information).

### Characterization of Wrinkle Wavelength and Amplitude

Wrinkle wavelength and amplitude were observed to be affected by trapped strain, film type, film thickness, and number of orthogonal layers. At both low and high trapped strains, Au wrinkle wavelength and amplitude were observed to significantly increase as film thickness increased (Figure 6). In the same samples, wavelength increased, while amplitude decreased as the number of orthogonal layers increased (Figure 6). Also, simple wrinkles were observed to have smaller wavelengths and larger amplitudes than the complex 2D postbuckling patterns (Figure 6).

Conversely, in the PS group, the effect of orthogonal layers and film thickness on wrinkle wavelength and amplitude was observed to be strain dependent. At high trapped strains, PS wrinkles behave like Au wrinkles (Figure 7A). As orthogonal layers increase, wrinkle wavelengths increase as the amplitude decreases (Figure 7A). At the same strains, PS wrinkle wavelength and amplitude significantly increased as the film thickness increased (Figure 7A). Also, simple wrinkles had a smaller wavelength and larger amplitude than complex 2D postbuckling patterns (Figure 7A). At lower trapped strains, on the other hand, orthogonal layers had no clear effect on wrinkle wavelength since wavelength appears the same irrespective of orthogonal layer (Figure 7B­(i)) while amplitude was observed to decrease as the number of orthogonal layers increased (Figure 7B­(ii)). In the same strain condition, wavelength increased as film thickness increased (Figure 7B­(i)); however, film thickness had no clear effect on amplitude; in various orthogonal layer conditions, amplitude increased, decreased, or remained the same as film thickness increased (Figure 7B­(ii)).

**Figure 7:**
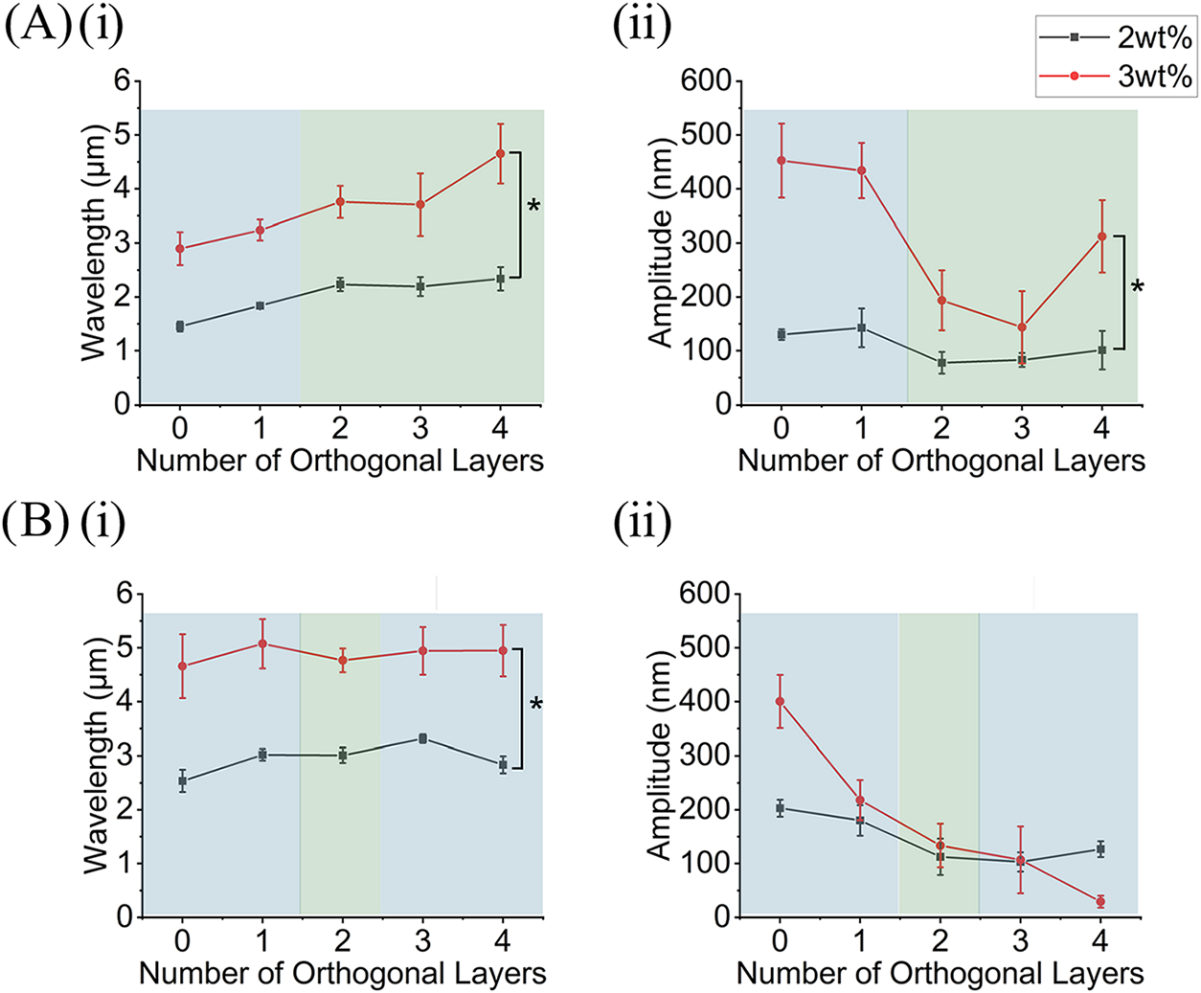
Wavelength and amplitude of PS wrinkles. (A)­(i) Wavelength and (ii) amplitude of PS wrinkles on the surface of substrates printed at 200 °C. (B)­(i) Wavelength and (ii) amplitude of PS wrinkles on the surface of substrates printed at 220 °C. The blue highlight denotes simple wrinkle regime, and the green highlight denotes the complex 2D postbuckling patterns regime. Each point corresponds to an average of three samples and the error bars represent their standard deviation. All data were compared using one-way ANOVA with Holm–Sidak post hoc test (*p < 0.05).

The relationship between wrinkle dimensions (wavelength and amplitude) and film thickness and film type follows reported theoretical expectations. Theoretical analysis of wrinkle behavior using linear buckling theory^4,7^ or the finite deformations model^25^ show that film thickness is proportional to wavelength and amplitude. All results except the amplitude of PS wrinkles on the surface of the 220 °C printed samples showed a statistically significant dependence on the film thickness. As noted above, the inconsistent behavior of the low-strain (high nozzle temperature) samples can be attributed to the critical strain. Lastly, the effect of film type on wrinkle behavior according to theory is due to the substantial difference in the Young’s moduli and thicknesses of Au and PS. Spin coating of PS produces thicker films than sputter coating of Au (Figure S1, see Supporting Information).

### Cytocompatibility of the Surface Patterns

The characterization of wrinkle morphology and dimensions sets the stage for the evaluation of the cellular response to the surface patterns. Surface wrinkles have been shown as a cost-effective surface modifications strategy that allows the investigation of cell–matrix interactions.^4^ Surface wrinkles have been shown to affect stem cell differentiation,^26^ neuronal elongation,^27^ and neurogenesis.^28^ To allow future investigation of the biomedical relevance of the wrinkles presented in this study, we characterized the cytocompatibility of both the simple wrinkles and complex 2D postbuckling patterns.

Cells showed high viability (>90%) irrespective of wrinkle morphology 24 h post seeding (Figure 8B, *p* > 0.05). Qualitatively, the surface morphology of the platform on which the cells were seeded was observed to affect the cell morphology. In the control condition, cells seeded on flat tissue culture polystyrene spread out evenly and did not show any clear preferential orientation (Figure 8A­(i)). Cells seeded on simple wrinkles, on the other hand, aligned parallel to the wrinkle direction (Figure 8A­(ii)). Similar to the control, cells seeded on complex 2D postbuckling patterns did not exhibit a preferential orientation, but unlike the control condition, cells on complex 2D postbuckling patterns clustered together, forming cellular islands (Figure 8A­(iii)).

**Figure 8:**
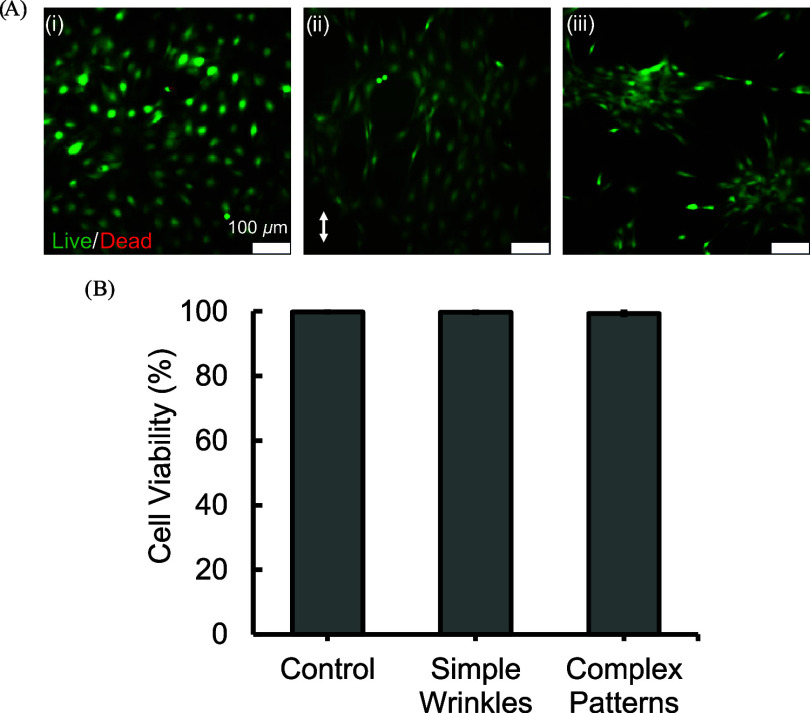
Cell viability of simple wrinkles and complex 2D postbuckling patterns. (A) C3H10T1/2 cells stained with calcein-AM (green) and ethidium homodimer (red). (A)­(i) Cells seeded on tissue culture polystyrene control. (A)­(ii) Cells seeded on simple wrinkles. The white double headed arrow denotes the wrinkle direction. (A)­(iii) Cells on the surface of complex 2D postbuckling patterns. (B) Percentage of cells viable. Each bar corresponds to an average of three samples, and the error bars represent their standard deviation.

The cytocompatibility of the complex 2D postbuckling patterns and their effect on cell morphology are important for future biomedical research. In previous studies, simple wrinkles were shown to direct nuclear orientation while being cytocompatible.^18^ By showing that the complex 2D postbuckling patterns are cytocompatible, this work sets the stage for the investigation of the mechanobiological relevance of the change in cell morphology induced by complex 2D postbuckling patterns.

## Conclusions

In this study, the introduction of orthogonal layers facilitated robust control over the direction of strain, producing unidirectional and bidirectional contractile strains. These strains were observed to affect wrinkle morphology, generating simple aligned wrinkles and complex 2D postbuckling patterns, respectively. We thoroughly characterized wrinkle dimensions as a function of strain (nozzle temperature and orthogonal layers) and film properties (film type and thickness), demonstrating agreement to theoretical expectations. These wrinkles were shown to be cytocompatible and capable of altering cell morphology, thus setting the stage for mechanobiological and biomedical studies.

Overall, we demonstrate an accessible and easily democratizable strategy that can produce cytocompatible simple wrinkles and complex 2D postbuckling patterns. Importantly, the approach presented uses a hobbyist 3D printer and Programming via Printing (PvP), a recently developed single-step SMP fabrication approach, providing an alternative to approaches that employ mechanical actuation achieved via costly or complicated uniaxial or multiaxial loading rigs. The wrinkles and postbuckling patterns demonstrated can be used as templates for soft lithography, suggesting that PvP can also complement conventional approaches.

## Supplementary Material



## Notes

Control of cytocompatible metallic and polymeric wrinkle morphologies using programming via printing (PDF)

## Abbreviations

SMP: shape-memory polymer
PvP: programming via printing
EBL: e-beam lithography
Au: gold
PS: polystyrene
AFM: atomic force microscope
wt %: weight percent
