# Supplementary material for: Control of Cytocompatible Metallic and Polymeric Wrinkle Morphologies Using Programming via Printing (PvP)
Source: ACS Omega. 2026 Jan 5;11(2):3326–35. doi: 10.1021/acsomega.5c10402 (PMC12824755; doi:10.1021/acsomega.5c10402)
Supplement: Supplementary file 1 [file ao5c10402_si_001.pdf]

Control of Cytocompatible Metallic and Polymeric Wrinkle Morphologies Using Programming via Printing (PvP)

Johnson N. Agyapong[1,3‡], Teng Zhang[1,2], and James H. Henderson[1,3‡]*

[1]Bioinspired Syracuse: Institute for Materials and Living Science, Syracuse University, Syracuse, NY 13244, USA

[2]Department of Mechanical and Aerospace Engineering, Syracuse University, Syracuse, NY 13244, USA

[3]Department of Biomedical & Chemical Engineering, Syracuse University, Syracuse, NY 13244, USA

QUANTITATIVE REPRESENTATION OF WRINKLES

The wavelength and amplitude analysis of the highly deformed wrinkles

$$\lambda_0 = 2\pi h \left(\frac{\bar{E}_f}{3\bar{E}_s}\right)^{1/3} \text{and } A_0 = h\sqrt{\frac{\epsilon_r}{\epsilon_c} - 1}$$

where, $\bar{E}$ represents the plane strain modulus, which is related to the Young's modulus (E) and the Poisson's ratio (v) as follows $\bar{E} = E/(1 - v^2)$. $\bar{E}_s$ and $\bar{E}_f$ represent substrate and film, respectively, ϵ_r is the recovery strain. At the final deformation, the wavelength and amplitude should be updated as

$$\lambda = \lambda_0 = 2\pi h \left(\frac{\bar{E}_f}{3\bar{E}_s}\right)^{1/3}$$

CHARACTERIZATION OF FILM THICKNESS

It was observed that film thickness was affected by sputter time for Au films and weight percent for PS film. As sputter time or weight percent increased, film thickness increased as well. The PS films were also thicker than the Au.

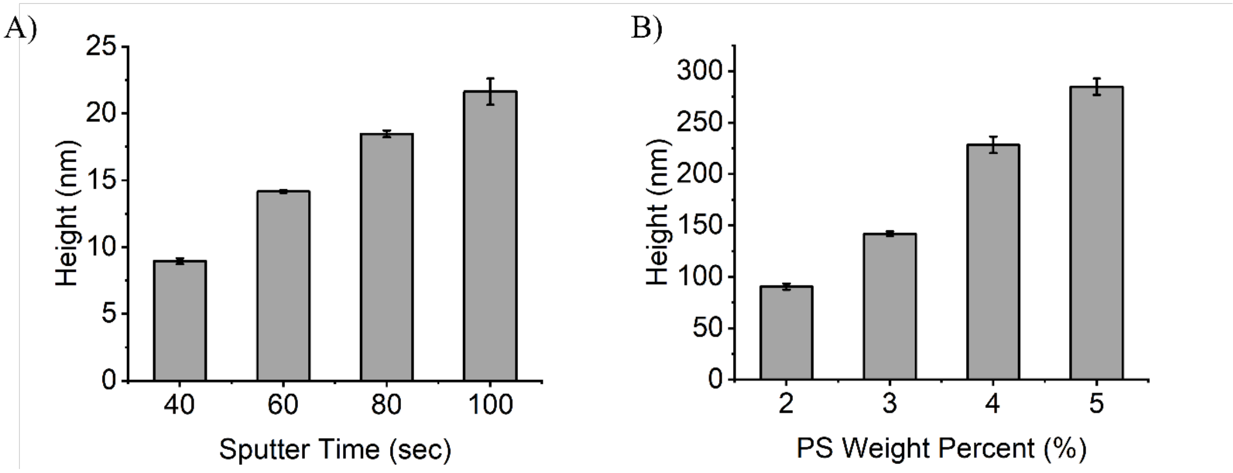

Figure S1: Film thickness was controlled by modifying A) Au sputter time and B) PS weight percent. As Au sputter time and PS wt% increased, film thickness increased as well. During sputtering, the power was set to 45 mA and kept constant to ensure that the amount of Au deposited is controlled by time. In the PS condition, the PS volume, spin coater spin rate, time and acceleration were kept constant for all weight percents to ensure that PS concentration was the main parameter that affect PS thickness. Each data point was produced from three technical replicates, and the error bars represent the standard deviation.

WRINKLE MORPHOLOGIES AT THE MACROSCOPIC LEVEL

Digital microscope images of the printed substrates showed that wrinkle morphology was affected by the introduction of orthogonal layers. Using the Au samples printed at 200 ℃ (high trapped strain) and sputtered for 100 s as an example, the effect of orthogonal layers on wrinkle morphology at the macroscopic level becomes apparent. At zero orthogonal layers, simple aligned wrinkles with cracks (Fig. S2 row A column 0) running perpendicular to the wrinkles were observed. When one orthogonal layer was introduced, the wrinkle morphology continued to be simple but there were less cracks (Fig. S2 row A column 1). The wrinkle morphology was observed to change at two orthogonal layers and above. The complex wrinkles became more discernible at four orthogonal layers (Fig. S2 row A column 4).

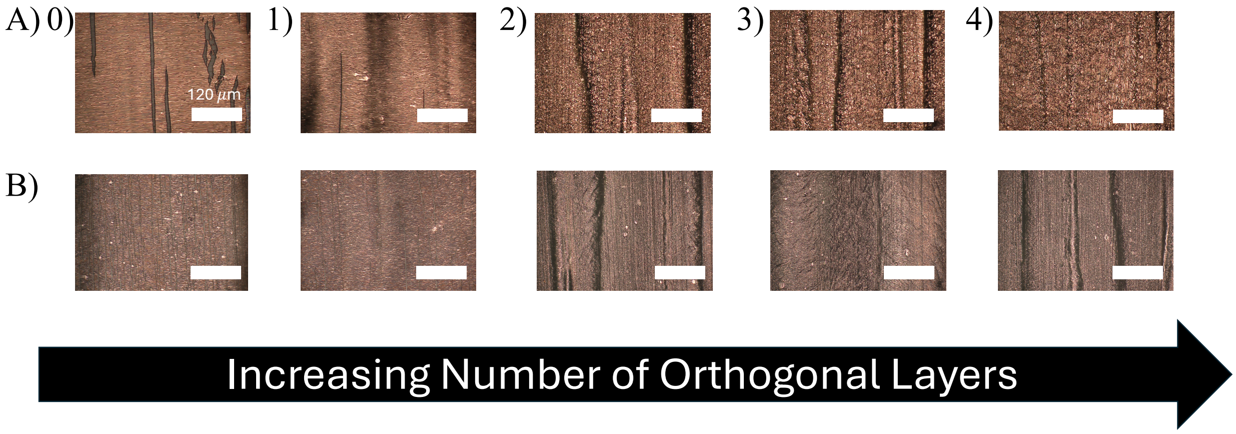

Figure S2: Digital microscope images of wrinkled Au of different thickness on the surface of substrates printed at 200 °C. (A) Substrates sputtered for 100 s which yielded a thickness of 21.63 ± 0.98 nm. (B) Substrates sputtered for 40s which yielded an Au thickness of 8.95 ± 0.21 nm. The depicted substrates were composed of zero (0), one (1), two (2), three (3), or four (4) orthogonal layers. The vertical streaks on are cracks whereas the vertical hills and valleys across the some of the images are the welding points between the extruded filaments.

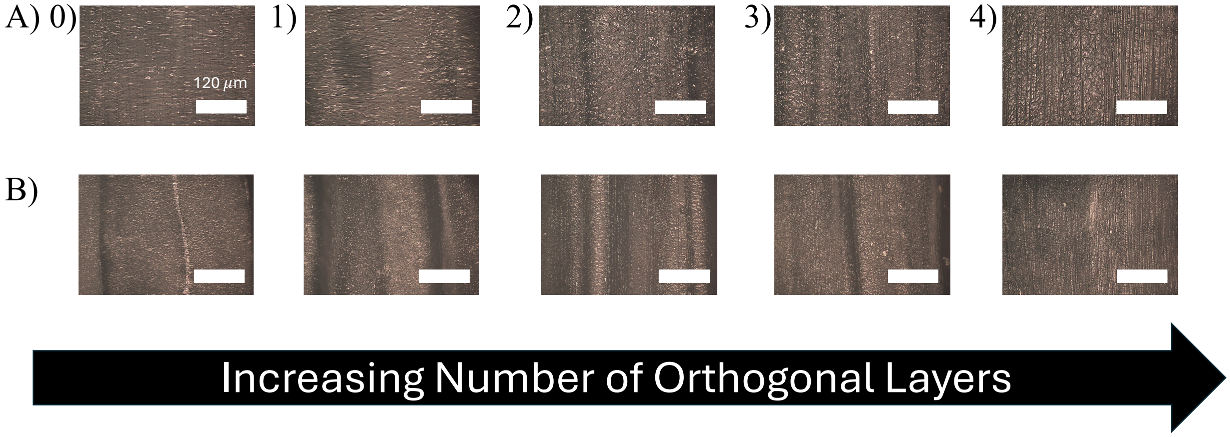

Figure S3: Digital microscope images of wrinkled PS on the surface of substrates printed at 200 °C. (A) Substrates coated with 3 wt% PS which yielded a PS thickness of 284.92 ± 7.88 nm. (B) Substrates coated with 2 wt% PS which yielded a PS thickness of 90.47 ± 2.95 nm. The depicted substrates were composed of zero (0), one (1), two (2), three (3), or four (4) orthogonal layers. The vertical hills and valleys across the some of the images are the welding points between the extruded filaments. The filaments within the surface of the substrates represented in this figured were extruded along the vertical direction.

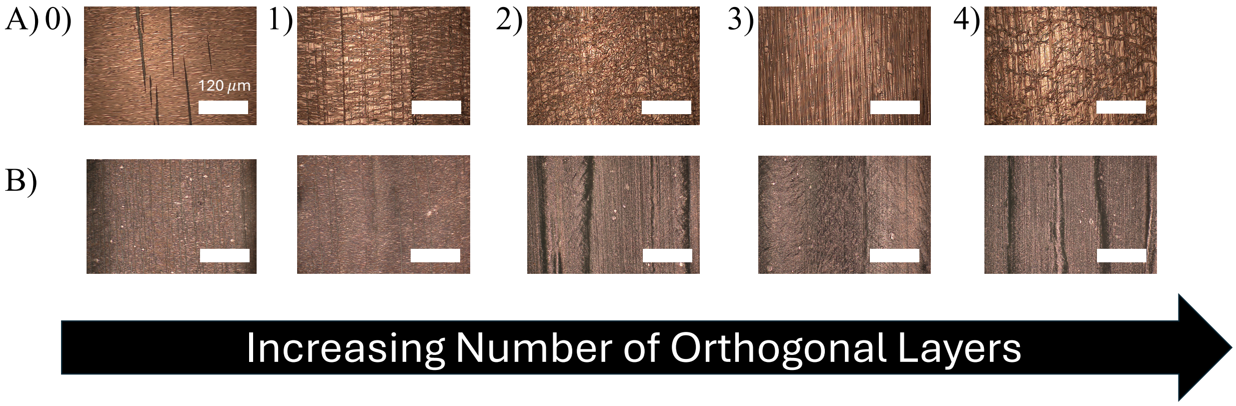

Figure S4: Digital microscope images of wrinkled Au of different thicknesses on the surface of substrates printed at 220 °C. (A) Substrates sputtered for 100 s which yielded a thickness of 21.63 ± 0.98 nm. (B) Substrates sputtered for 40 s which yielded an Au thickness of 8.95 ± 0.21 nm. The depicted substrates were composed of zero (0), one (1), two (2), three (3), or four (4) orthogonal layers.

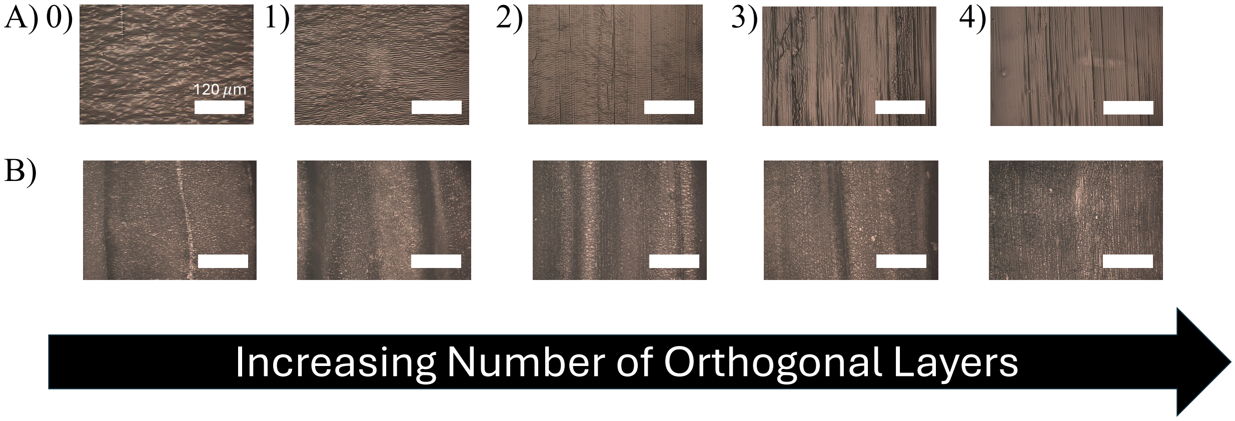

Figure S5: Digital microscope images of wrinkled PS of different thicknesses on the surface of substrates printed at 220 °C. (A) Substrates coated with 3 wt% PS which yielded a PS thickness of 284.92 ± 7.88 nm. (B) Substrates coated with 2 wt% PS which yielded a PS thickness of 90.47 ± 2.95 nm. The depicted substrates were composed of zero (0), one (1), two (2), three (3), or four (4) orthogonal layers.

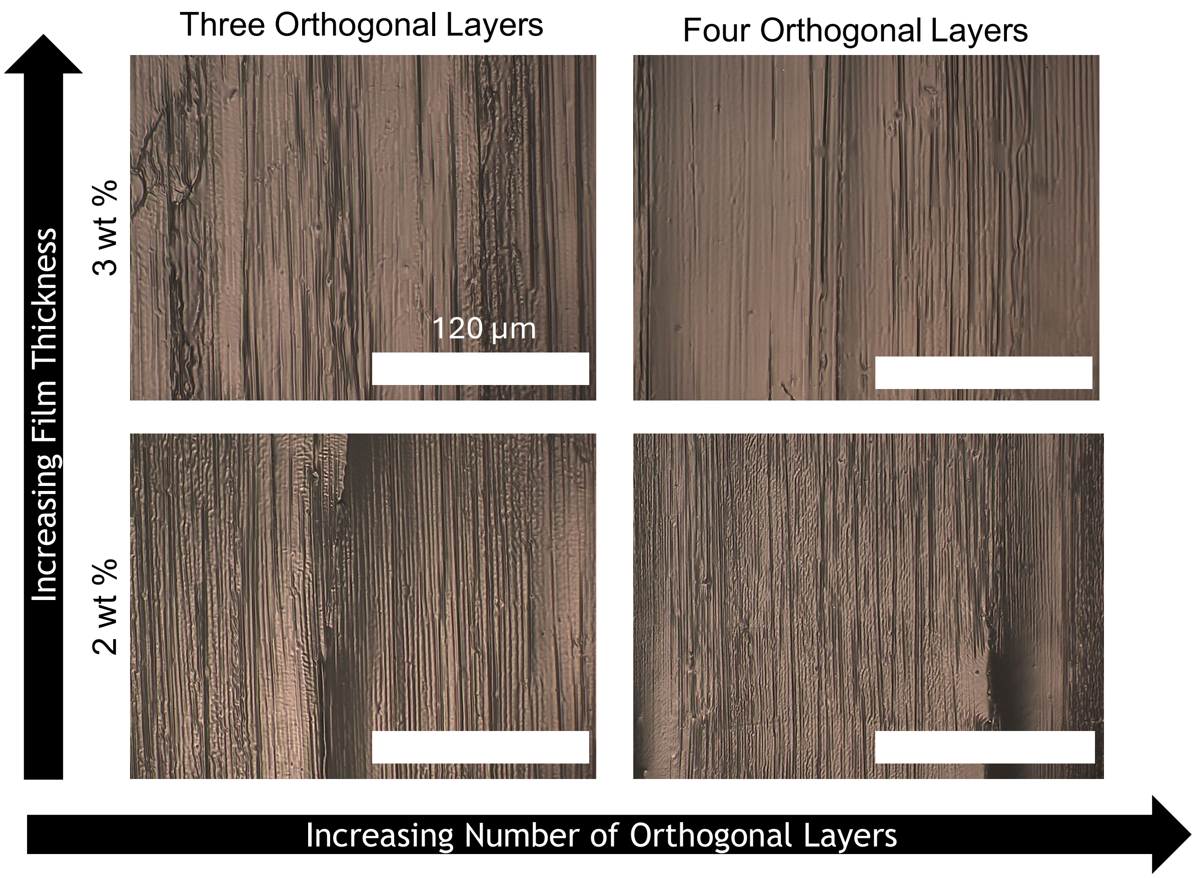

Figure S6: Digital image of PS wrinkles with smooth regions on the surface of sample printed at 220 °C. The samples displayed were composed of three or four orthogonal layers. The thinner film in this image was coated with 2 wt% PS which produced a 90.47 ± 2.95 nm film. The thick film in this image was coated with 3 wt% PS which produced a 284.92 ± 7.88 nm film.
